# Remarks on Mr. Bishopp's Case of Rheumatism

**Published:** 1805-10-01

**Authors:** E. Harrold

**Affiliations:** Cheshunt, Herts


### *To the Editors of the Medical and Physical Journal.*

Gentlemen,

When the nature of a disorder and the effect of remedies in its treatment are perfectly clear, however unusual the disease, and however new the practice, conciseness is always agreeable to the reader. But when there is any doubt respecting its nature, and the effect of remedies is uncertain or unsuccessful; if the case be submitted to public opinion, its history should be as full and particular as possible. For it too commonly happens, that from some of the more striking symptoms the practitioner makes up his mind as to the nature of the case, and pays little attention to other symptoms, which, in his view of it, may appear either subordinate or anomalous. In such circumstances, if he were to write its history concisely, he would describe only what *appeared to him* to be its characteristic marks. Such a narrow view of the subject would, of course, be of little use to the medical world, and perhaps serve only to show, unfairly indeed, the uselessness, on some occasions, of powerful remedies. This early impression of the nature of a disease has very often more influence upon the mind of the practitioner than many would be willing to allow, more especially after its nosological name is fixed, and sometimes prevents his seeing what, to a new observer, would be sufficiently obvious. This is one of the best reasons for calling in the aid of a physician, and sometimes of a second; and it might be useful in the progress of an obscure and untractable disorder, to pause, and ask ourselves, what would probably be the opinion of another at that particular moment? There is, I presume, no medical man who has not sometimes wished, on a retrospective view of a disease which has terminated unfavourably, that he had pursued a different mode of treatment.

The case of Thomas Ains, recorded in the 78th Number of the Medical and Physical Journal, by Mr. Bishopp, is very interesting; but it would have been more satisfactory could Mr. B. have ascertained by dissection, what was the cause of the dyspnœa he mentions; whether it was hydrothorax, or abscess, or mere debility. It is an uncommon case which he has given to the world; no doubt, for the purpose of discussion; and as his mode of treatment was the same that most medical men perhaps would employ, he will, I am sure, be pleased at a few general remarks.

We all know that there are many diseases which affect the whole vascular system, whose progress seems not to be at all interrupted by depletion. This is remarkably the case in some kinds of fever, it is not unfrequently so in some inflammatory affections, as in the case of Ains, where the morbid vascular action continued until he was "completely exhausted," in spite of "topical and general blood-letting, relaxants, &c. It therefore appears that inflammatory action does not acquire a full habit as a necessary condition to its existence, and, when the abstraction of blood proves curative, that it does so in an indirect manner. The abstraction of blood may be considered dered in two points of view, as it relates to the mechanical effect of taking away a part of the weight propelled by the action of the heart and arteries, and as it relates to its influence upon the nervous system through the new condition of the balance between the solids and fluids. If it were not for the latter consideration we should be unable to account for the difference of effect produced by the abstraction of a given quantity of blood from a large and from a small orifice, the one effecting the nervous excitability more than the other. There is an idea entertained by the common people, that the first bleeding must be useful to them in a remarkable degree, and therefore many of them keep this remedy in reserve for some very important occasion. If the above reasoning be true, it affords some slight support to this vulgar prejudice; the remedy too may be more powerful from such an opinion of its efficacy. Where the blood vessels are really over-powered by *quantity* of blood, it constitutes a disease for which the loss of blood acts as a direct remedy; but in those inflammatory diseases where its abstraction is sometimes, but not always curative, I apprehend that it acts as an indirect remedy, affecting the nervous system principally. Therefore, when in such cases it has been carried to a certain extent without disturbing the morbid action, I should expect no good effect from its farther use, but rather that it would hasten the fatal termination of the disease. I have seen blood-letting employed to a considerable extent in acute rheumatism, without the expected advantage. The blood indeed is generally more sizy at the second and third bleeding than at first. By its use (except perhaps in strong and full habits at the outset) the strength of the patient is commonly diminished, but not the violence of the disorder. I have found the disease yield much more certainly and speedily to powerful doses of cinchona, with opium at night; and the sooner this plan is adopted, after the tongue becomes moist and clean round its edge, the sooner will the patient be cured. It commonly increases the pain at first; but if employed sufficiently early, the patient is usually well in a fortnight, and not extremely debilitated and so subject to relapse as by the common mode of management.

In bleedings from the nose, where the hæmorrhage has been large and frequent, there are still found marks of febrile action; a white tongue; a large, quick, and frequent pulse; but which will not, in general, raise the finger very powerfully. In this case, a single bleeding from the arm is sometimes useful, producing what has been called a re-

 vulsion, vulsion, but what I should call its indirect effect. Where the hæmorrhage has been (as above stated) large and frequent, there cannot be much plethora, and I should consider a repetition of venæsection bad practice. In such cases (and I have seen many) I have never known large doses of cinchona with tinct. opii (for instance, ℨj. of the former and gtt. x of the latter, 6 horis, according to the age, &c. of the patient) to fail in curing very speedily and very permanently, if continued a sufficient length of time. I am disposed to think, that any great tendency to suppurative inflammation rather indicates want of tone; this is frequently seen in irritable habits, where whitlows sometimes affect all the fingers. Sometimes there is a disposition in the habit to produce suppurating boils on almost every part of the skin; a very painful and troublesome complaint, but which, as well as the former, is sooner cured by large doses of cinchona, as far as my experience goes, than by any other mode of cure. These facts are stated, not merely as they relate to the respective complaints, but as a ground for the trial of the same plan in cases of suppurative rheumatism; for if it were adopted before any vital part is affected, it might destroy the suppurative habit. Here the question again occurs, what was the state of the thoracic viscera in the case of Ains?

I have formerly seen in Hospital practice, an unconquerable tendency to hæmorrhage after amputation, where the hæmorrhagic nisus continued till the patients were exhausted. Here I should expect the same effect from cinchona and opium as in epistaxis.

As I have no other design, Gentlemen, in sending you the foregoing Observations, than that of contributing to the stock of useful medical knowledge; if you think them calculated to answer that purpose, you will do me the favour to insert them in your very useful Journal.

I am, &c.

*Cheshunt, Herts, Sept.* 2, 1805. E. HARROLD.

P. S. Since writing the above, I have seen your "Critical Analysis" of Dr. Haygarth's Clinical Work; a work which I have not yet had the good fortune to see. My communication can of course, as far as it relates to rheumatism, have no pretension to originality; but in so far as it may prove a humble support to the highly reputable testimony of Dr. Haygarth, it may be thought more worthy of attention. That the early use of cinchona in acute rheumatism has been my practice for near thirteen years, some of my friends and many of my patients could testify.

